# Pericapsular Nerve Group (PENG) Block Versus Suprainguinal Fascia Iliaca Block (SI-FICB) for Perioperative Analgesia in Hip Fracture Surgery: A Systematic Review and Meta-Analysis

**DOI:** 10.7759/cureus.98632

**Published:** 2025-12-07

**Authors:** André Fernandes, Charlotte MacAulay, Natalia Chamberlain, Chen Y Ooi, Heather Tetley, Shaul Gordon, Marcus Cox, Oliver Dixon, Hany Saleeb

**Affiliations:** 1 Trauma and Orthopedics, York and Scarborough NHS Trust, York, GBR; 2 Trauma and Orthopedics, Hull York Medical School, York, GBR

**Keywords:** elderly patients, hip fracture, meta-analysis, neuraxial anaesthesia, pericapsular nerve group block (peng), peri-operative analgesia, randomized controlled trial, regional anaesthesia, supra-inguinal fascia iliaca block (si-ficb), systematic review

## Abstract

The optimal regional anesthetic technique to facilitate spinal anesthesia and early mobilization in hip fracture surgery remains uncertain. The suprainguinal fascia iliaca block (SI-FICB) aims to enhance cranial spread within the fascia iliaca plane, whereas the pericapsular nerve group (PENG) block selectively targets articular branches to the anterior hip capsule with a motor-sparing intent. Randomized trials comparing the two techniques have produced heterogeneous results. We conducted a systematic review and meta-analysis of randomized controlled trials directly comparing PENG with SI-FICB in adults (≥60 years) undergoing operative management of acute hip fracture under neuraxial anesthesia. The primary outcome was pain within 60 minutes of block administration, with secondary outcomes including pain at rest, pain on movement, time to first rescue analgesia, number of rescue doses, and 24 h opioid consumption. Continuous data were synthesized using random-effects modelling (REML) with Hartung-Knapp-Sidik-Jonkman adjustment, and medians with IQRs or ranges were transformed to mean±SD using the Wan and Luo methods. Risk of bias was assessed using RoB 2 and certainty of evidence using the Grading of Recommendations Assessment, Development and Evaluation (GRADE). Eleven perioperative RCTs (n=1,260; PENG: 630; SI-FICB: 630) were included. PENG reduced pain at rest within 60 minutes (mean difference {MD} -0.51, 95% CI: -0.74 to -0.28; T=4.97; I²=81%) and pain on movement (MD: -0.52, 95% CI: -0.71 to -0.34; T=6.49; I²=0%). No significant differences were observed for time to first rescue (MD -0.83 h, 95% CI: -1.92 to 0.26; T=1.75; I²=95%), number of rescue doses (MD: -0.08, 95% CI: -0.46 to 0.29; T=0.71; I²=37%), or 24 h opioid consumption (MD: -15.59 mg oral morphine equivalent {OME}, 95% CI: -43.00 to 11.83; T=1.58; I²=100%). The “T” value represents the test statistic produced by the Hartung-Knapp-Sidik-Jonkman (HKSJ) adjustment in the random-effects model. PENG provides superior immediate analgesia for spinal positioning and early perioperative pain control compared with SI-FICB, though heterogeneity in secondary outcomes highlights the need for further standardized RCTs to clarify optimal dosing, timing, and functional endpoints.

## Introduction and background

A hip fracture is a time-critical emergency in older adults, and effective perioperative analgesia is essential to facilitate neuraxial anesthesia and early mobilization. Hip fractures are associated with substantial morbidity and mortality, with 30-day mortality approaching 10% and one-year mortality exceeding 20%, much of which is driven by pain-related immobility, delirium, pneumonia, and venous thromboembolism [1]. High-quality analgesia supports patient comfort, improves positioning for spinal anesthesia, reduces perioperative opioid requirements, and contributes to early rehabilitation, which is a key priority in contemporary hip fracture pathways [2,3].

The fascia iliaca compartment block is well established for proximal femoral and hip fracture pain, and early descriptions demonstrated a simple and reliable alternative to femoral and 3-in-1 blocks [4]. With the introduction of ultrasound guidance, a suprainguinal modification was developed to promote cephalad spread within the fascia iliaca plane toward the lumbar plexus, improving access to the femoral, obturator, and lateral femoral cutaneous nerves, and enhancing coverage for hip pathology [5]. Suprainguinal fascia iliaca block has been increasingly incorporated into perioperative practice because of its broader plexus coverage and its ability to facilitate pain-free positioning for spinal anesthesia.

In 2018, the pericapsular nerve group block was described as a targeted articular block for analgesia in hip fractures. The technique deposits local anesthetic adjacent to the sensory branches supplying the anterior hip capsule, predominantly arising from the femoral and accessory obturator nerves [6]. This approach was supported by anatomical work demonstrating that the anterior hip capsule is the principal driver of nociception in hip pathology [7]. The pericapsular nerve group block, therefore, carries a theoretical advantage of providing strong analgesia while sparing quadriceps strength, which may improve early mobilization and reduce perioperative motor impairment.

Since 2021, multiple randomized controlled trials have compared the pericapsular nerve group block with fascia iliaca techniques in hip fracture populations. The expansion of these trials reflects clinical uncertainty regarding whether a more selective articular block can outperform a well-established field block in perioperative practice. Earlier reviews often pooled infrainguinal fascia iliaca approaches, mixed surgical and non-operative cohorts, or included emergency department studies where timing and analgesic objectives differ from operative care [8,9]. These limitations reduce the relevance of previous analyses to the perioperative environment.

The aim of this review was to synthesize perioperative evidence from randomized controlled trials that directly compared the pericapsular nerve group block with the suprainguinal fascia iliaca block for adults undergoing hip fracture surgery. Outcomes relevant to anesthetic practice were prioritized, including immediate post-block pain scores, dynamic pain, the timing of rescue analgesia, and early opioid exposure. Clarifying the comparative effectiveness of these two widely used techniques may help inform clinical decision-making and support optimized hip fracture anesthesia pathways.

## Review

Methods

This systematic review was conducted in accordance with the Cochrane Handbook for Systematic Reviews of Interventions and is reported following the Preferred Reporting Items for Systematic Reviews and Meta-Analyses (PRISMA) 2020 statement. The protocol was registered on PROSPERO (#CRD420251179710), prior to final submission [10,11]. Risk of bias was evaluated using the Cochrane risk of bias 2 (RoB 2) tool, and the certainty of evidence for each critical outcome was assessed using the Grading of Recommendations Assessment, Development and Evaluation (GRADE) framework.

Eligible studies included parallel-group randomized controlled trials that enrolled adults aged 60 years or older with acute hip fracture, including femoral neck, intertrochanteric, and subtrochanteric subtypes, undergoing operative fixation or arthroplasty under spinal anesthesia. Exclusion criteria were femoral shaft or pathological fractures, pediatric populations, prehospital or emergency department interventions, and non-operative management. Trials comparing infrainguinal or classic fascia iliac techniques, or other regional anesthetic modalities, were excluded.

The intervention of interest was a single-shot, ultrasound-guided pericapsular nerve group (PENG) block administered perioperatively, either on the ward or in theatre before spinal anesthesia or surgical incision, using 0.25% to 0.5% bupivacaine or ropivacaine in volumes of 20-30 mL. The comparator was an ultrasound-guided suprainguinal fascia iliaca compartment block (SI-FICB) using comparable local anesthetic types, concentrations, and volumes.

The primary outcome was pain intensity measured on a visual analog scale (VAS) or numerical rating scale (NRS) from 0 to 10, assessed during positioning for spinal anesthesia or within 60 minutes after block administration. Secondary outcomes included pain at 2-24 h, time to first rescue analgesic, number of rescue doses within 24 h, and total 24 h opioid consumption expressed as oral morphine equivalent (OME).

A comprehensive search of MEDLINE via PubMed, Embase via Ovid, and the Cochrane Central Register of Controlled Trials (CENTRAL) was undertaken from January 2018, the year the PENG block was first described, to October 22, 2025. Search terms included controlled vocabulary and free-text keywords for “pericapsular nerve group block,” “suprainguinal fascia iliaca block,” “hip fracture,” “perioperative care,” “neuraxial anesthesia,” and “randomized controlled trial,” with filters excluding animal studies and prehospital or emergency department contexts. Reference lists of included trials and relevant systematic reviews were manually screened to identify additional eligible studies. The full PubMed search strategy is available in the appendix.

Study Selection and PRISMA Counts

**Figure 1:**
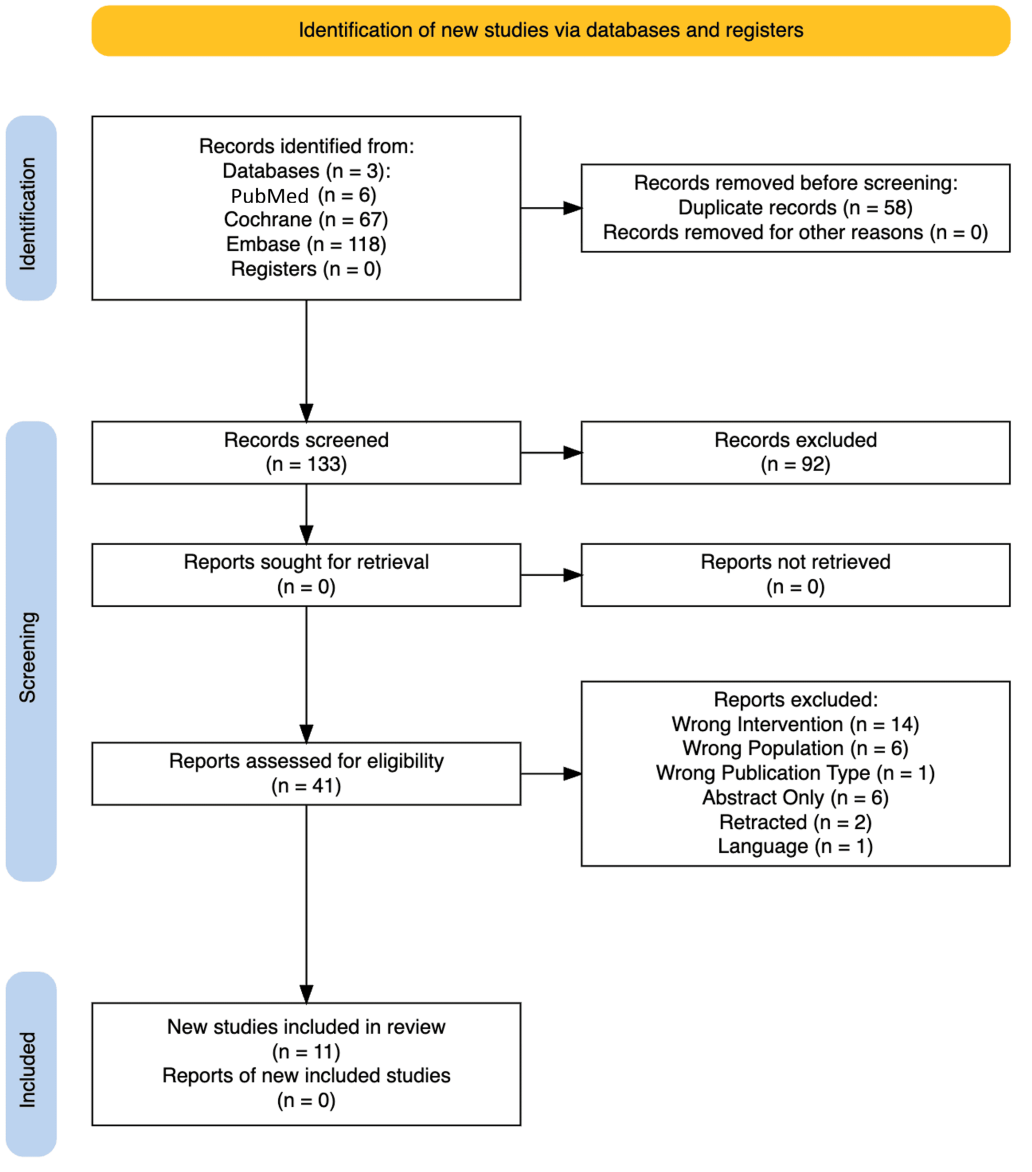
PRISMA flow diagram illustrating the study selection process. PRISMA: Preferred Reporting Items for Systematic Reviews and Meta-Analyses

The database search identified 191 records (PubMed, n=6; Cochrane, n=67; and Embase, n=118). After removing 58 duplicates, 133 unique titles and abstracts were screened, of which 92 were excluded. Forty-one full texts were assessed in detail, and 30 were excluded for reasons including wrong comparator, ineligible population, abstract-only publication, non-randomized or quasi-randomized design, retracted or redacted status, or unresolved suprainguinal versus infrainguinal classification despite author contact. Eleven randomized controlled trials met the inclusion criteria and were included in the qualitative and quantitative synthesis (Figure 1). Outcomes of interest extracted for quantitative synthesis included all prespecified primary and secondary endpoints suitable for pooled analysis. The primary outcome was pain intensity at rest within 60 minutes of block administration or during positioning for spinal anesthesia. Secondary outcomes comprised pain on movement within 24 h, time to first rescue analgesic, the number of rescue doses within 24 h, and total 24 h opioid consumption expressed as oral morphine equivalents. Safety outcomes, including postoperative nausea or vomiting and block-related complications, were extracted when reported, although low event rates precluded formal pooling.

Data Extraction and Transformation

Two reviewers independently extracted study-level data, including setting, randomization and blinding methods, local anesthetic type and volume, and timing of block administration. Outcome time-points and numerical results were recorded for all relevant measures. When data were presented as medians with interquartile ranges or ranges, means and standard deviations were estimated using the methods proposed by Wan et al. and Luo et al. [12,13]. Opioid doses were converted to oral morphine equivalents using study-specific conversion factors to standardize comparisons across trials.

Risk of Bias and Certainty

**Figure 2:**
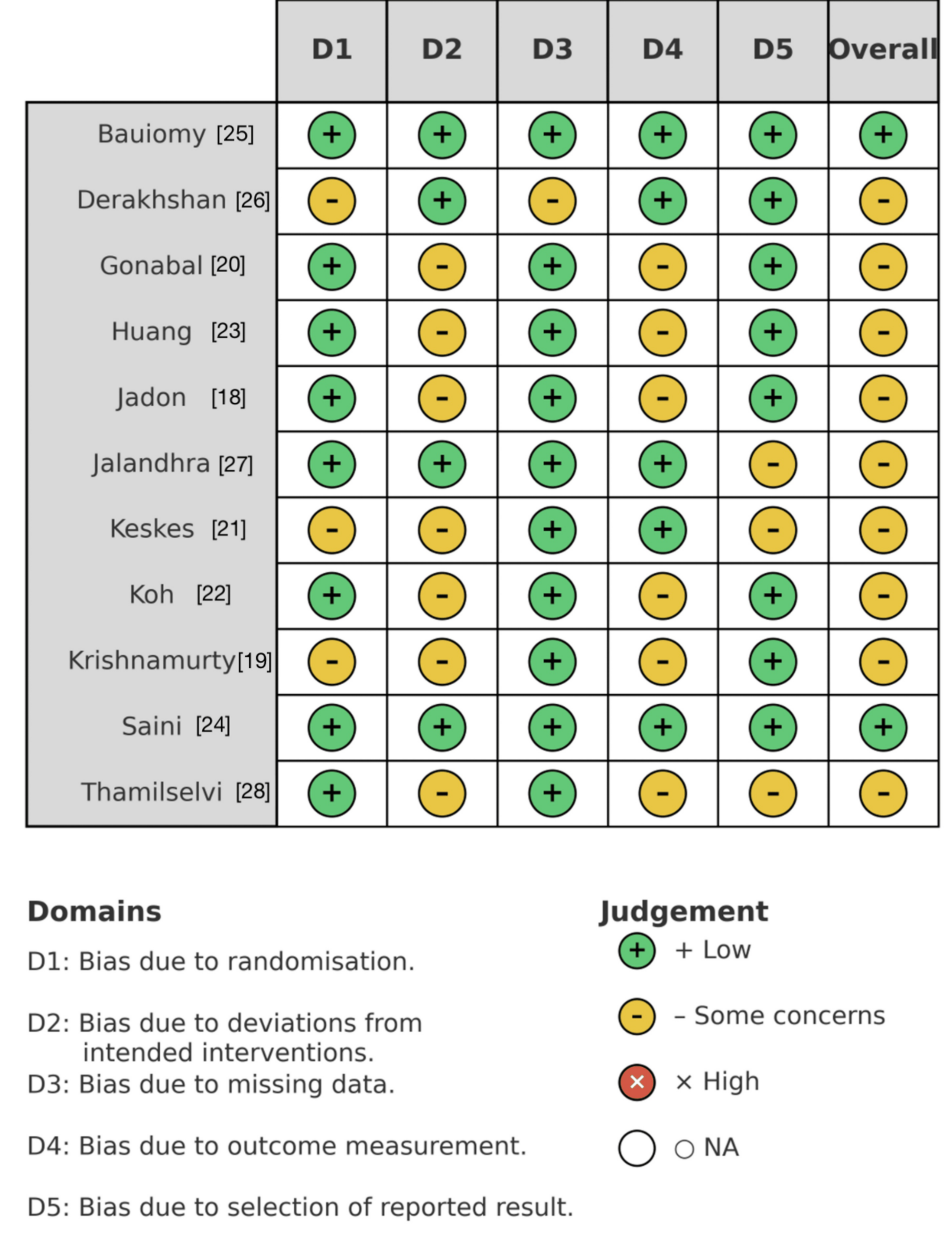
Risk of bias 2 (RoB 2) traffic-light plot for individual domains.

Risk of bias across the 11 included randomized controlled trials was assessed using the Cochrane risk of bias 2 (RoB 2) tool at the outcome level (Figure 2). The overall methodological quality of the evidence base was acceptable, with most studies demonstrating low to moderate risk across domains.

Random sequence generation and allocation concealment (Domain 1) were rated low risk in 10 studies and some concerns in one study, typically due to limited detail on envelope handling or sequence generation. Deviations from intended interventions (Domain 2) showed the greatest variability, with eight studies rated as some concerns, primarily because blinding of participants or anesthetists was infeasible during regional block placement. Nevertheless, all trials maintained blinding of postoperative assessors, mitigating potential bias in outcome measurement.

Bias due to missing outcome data (Domain 3) was low risk across all trials, as follow-up to 24 h was complete or nearly complete. Outcome measurement (Domain 4) was low risk in nine studies and some concerns in two, where pain assessment timing or assessor blinding was unclear. Selective reporting (Domain 5) was low risk in seven studies and some concerns in four, mainly due to incomplete trial registration or omission of prespecified secondary endpoints. No trial demonstrated a high risk of bias in any domain.

**Figure 3:**
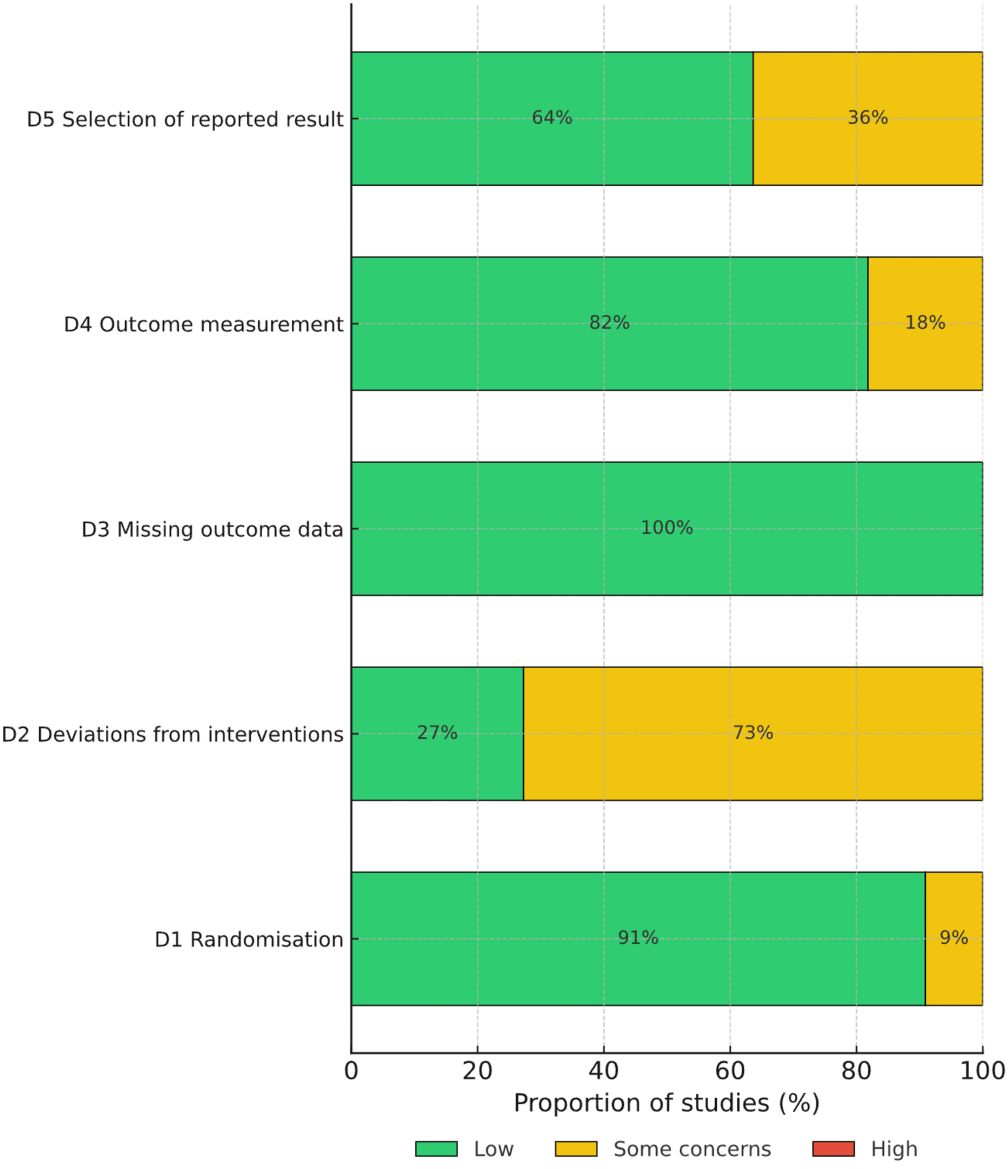
Summary of domain-level risk of bias across included studies.

A domain-level summary illustrates that randomization and data completeness were consistently robust, whereas performance-related bias and reporting transparency represented the most frequent limitations (Figure 3). The cumulative risk pattern supports a moderate overall confidence in the pooled effect estimates for the primary and secondary outcomes.

**Figure 4:**
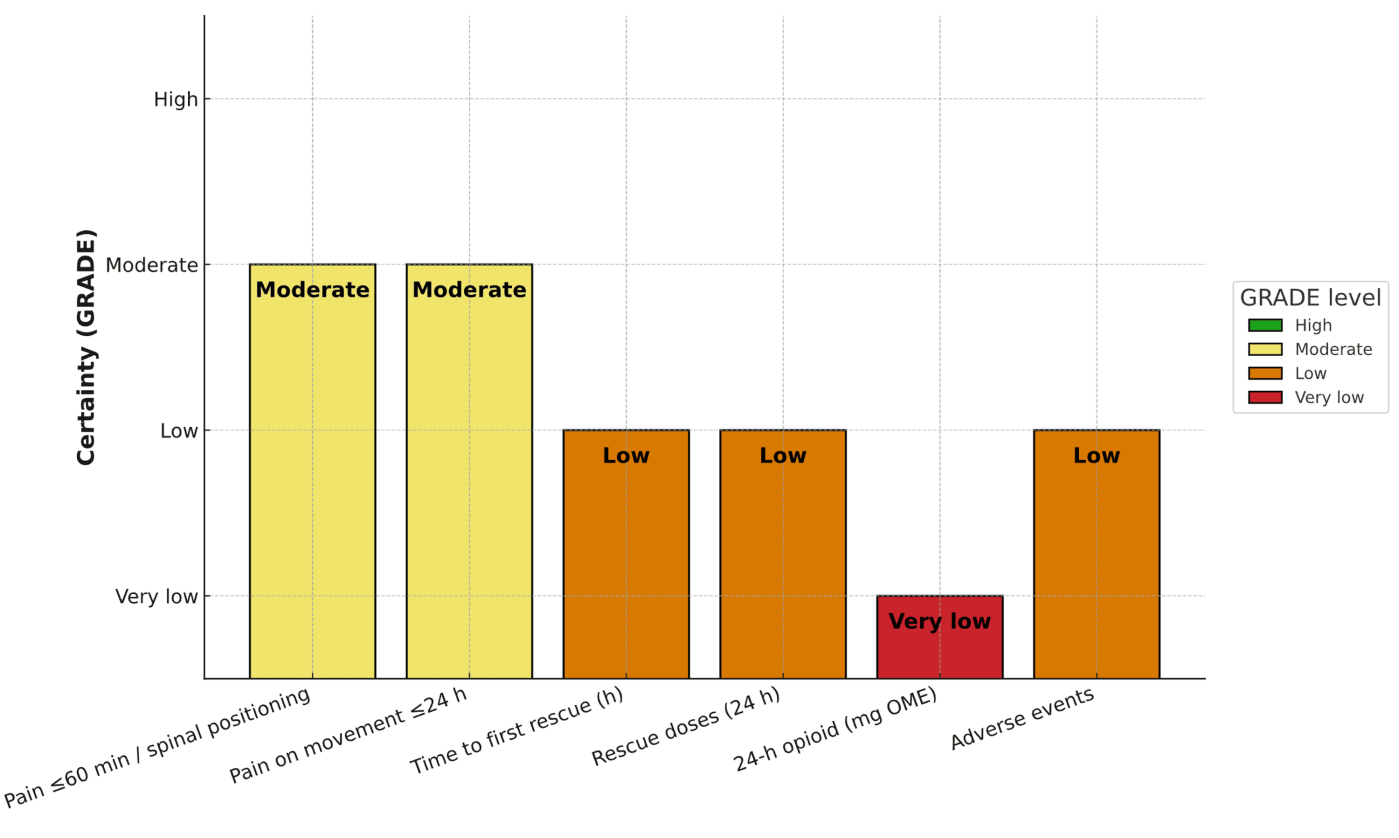
GRADE summary of findings - certainty of evidence for primary and secondary outcomes. GRADE: Grading of Recommendations Assessment, Development and Evaluation; OME: oral morphine equivalent

In alignment with GRADE certainty ratings, the primary outcomes (pain at rest within 60 min or during spinal positioning, and pain on movement within 24 h) were judged to have moderate-certainty evidence, downgraded once for inconsistency or performance bias (Figure 4). Secondary outcomes, including time to first rescue analgesic, number of rescue doses, and 24 h opioid consumption, were rated low to very low certainty, primarily due to heterogeneity and imprecision of estimates. Safety outcomes, when reported, had low certainty, reflecting small event counts and underpowered adverse-event analyses.

Overall, these findings indicate that although minor methodological concerns exist in blinding and selective reporting, the evidence base provides a credible and internally consistent foundation for comparative conclusions between PENG and SI-FICB in the perioperative management of hip fracture surgery.

Effect Measures and Statistical Synthesis

Continuous outcomes were summarized as mean differences and pooled using an inverse-variance random-effects model with restricted maximum likelihood (REML) variance estimation and Hartung-Knapp-Sidik-Jonkman adjustment for confidence intervals [12-14]. Statistical heterogeneity was quantified using tau-squared, I-squared, and Cochran’s Q statistics. Ninety-five percent prediction intervals were planned when at least three studies contributed to an analysis [15,16]. Prespecified sensitivity analyses included leave-one-out testing and fixed-effect modelling to assess result robustness. Forest plots for each pooled analysis are presented separately. Analyses were performed in RevMan Web 5.4 (London, England: Cochrane Collaboration) [17].

Results

Study Characteristics

Eleven randomized controlled trials (n=1,260) compared the pericapsular nerve group (PENG) block with the suprainguinal fascia iliaca compartment block (SI-FICB) in adults undergoing surgery for acute hip fracture [13-23]. Most trials used ropivacaine 0.25-0.375% in volumes of 20-30 mL. Block placement was performed either on the ward or in theatre before spinal anesthesia. Randomization methods were computer-generated with sealed opaque envelopes, and most trials achieved double-blinding, with several using single-blind assessor designs. The included trials were conducted between 2021 and 2025 across India, Egypt, Iran, Taiwan, South Korea, and Tunisia (Table 1).

**Table 1:** Summary of included randomized controlled trials comparing the pericapsular nerve group (PENG) block and the suprainguinal fascia iliaca (SI-FICB) block in hip fracture surgery. Data represent study-level characteristics of randomized controlled trials comparing pericapsular nerve group (P) block and suprainguinal fascia iliaca compartment block (F) in patients undergoing hip fracture surgery. Values are expressed as mean±standard deviation (SD) unless otherwise specified. NR: not reported; OR: operating room; ED: emergency department; RCT: randomized controlled trial; SNOSE: sequentially numbered, opaque, sealed envelopes

Studies	Journal	Study design	Country	Time frame	Setting	Randomization method	Total (n)	PENG (n)	SI-FICB (n)	Age (years, mean±SD)	Male (%)	BMI (kg/m², mean±SD)	Local anesthetic
Jadon et al. (2021) [18]	Indian Journal of Anaesthesia	RCT, double-blind	India	2019-2021	Operating room	Computer-generated opaque envelopes	66	33	33	70.39±11.45 (P) 67.87±13.12 (F)	40.9	30.15±3.76 (P) 29.5±3.67 (F)	Ropivacaine 0.25%, 20 mL
Krishnamurty et al. (2022) [19]	International Journal of Health Sciences	RCT, double-blind	India	NR	Operating room	Simple random allocation	80	40	40	55.43±21.37 (P) 51.28±23.27 (F)	56.25	NR	Ropivacaine 0.25%, 20 mL
Gonabal et al. (2024) [20]	Journal of Anaesthesiology Clinical Pharmacology	RCT, double-blind	India	2020-2022	Operating room	Computer-generated block randomization	60	30	30	43.8±16.0 (P) 44.5±11.5 (F)	53	25.1±1.0	Ropivacaine 0.375%, 20 mL
Keskes et al. (2023) [21]	Panafrican Medical Journal	RCT, single-blind	Tunisia	NR	Operating room	Computer-generated single-blind	89	44	45	76.51±8.8	53.9	25.9±3.5	Ropivacaine 0.25%, 20 mL
Koh et al. (2025) [22]	Regional Anesthesia and Pain Medicine	RCT, double-blind	South Korea	NR	Operating room	Random sequence, blinded observer, opaque envelopes	79	40	39	75.6±11.0 (P) 76.9±12.4 (F)	21.5	23.4±3.6 (P) 22.6±3.2 (F)	Ropivacaine 0.375%, 20 mL
Huang et al. (2024) [23]	Injury	RCT, double-blind	Taiwan	NR	OR & ED	Computer-generated double-blinded opaque envelopes	91	45	46	76 (70-82) (P) 69 (63-79) (F)	46	23.7±4.1	Ropivacaine 0.375%, 20 mL
Saini et al. (2024) [24]	Panafrican Medical Journal	RCT, double-blind	India	2023-2024	Operating room	Computer-generated opaque envelopes	60	30	30	72.50±8.61 (P) 73.63±6.59 (F)	51.67	NR	Ropivacaine 0.375%, 20 mL
Bauiomy et al. (2024) [25]	Egyptian Journal of Anaesthesia	RCT, double-blind	Egypt	NR	Operating room	Computer-generated block randomization	60	30	30	65.23±5.73 (P) 64.73±6.36 (F)	71.6	30.53±4.4 (P) 30.28±3.5 (F)	Ropivacaine 0.375%, 20 mL
Derakhshan et al. (2024) [26]	J Cell Anesthesiology	RCT, double-blind	Iran	NR	Operating room	Computer-generated block randomization	50	25	25	65.9±10.8 (P) 61.5±14.3 (F)	50% (P) 45.9% (F)	NR	Ropivacaine 0.25%, 30 mL
Jalandra et al. (2025) [27]	International Journal of Current Pharmaceutical Review and Research	RCT, double-blind	India	2023-2024	Operating room	Computer-generated opaque envelopes	70	35	35	62.34±16.08 (P) 52.37±15.64 (F)	54.3	NR	Ropivacaine 0.375%, 20 mL
Thamilselvi et al. (2025) [28]	International Journal of Academic Medicine and Pharmacy	RCT, single-blind	India	2022-2024	Operating room	SNOSE method single-blind	80	40	40	50.38±10.99 (P) 48.55±11.17 (F)	56.25	23.98±1.23 (P) 24.52±0.31 (F)	Ropivacaine 0.375%, 20 mL

Primary Outcome: Pain at Rest Within 60 Min or During Spinal Positioning

**Figure 5:**
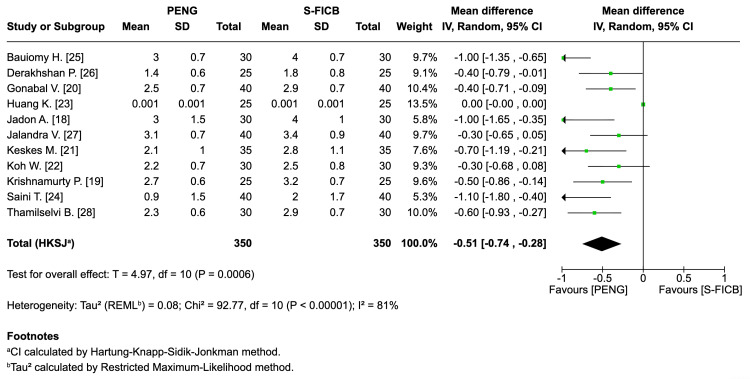
Forest plot - pain at rest within 60 min or during spinal positioning. REML: random-effects modeling; PENG: pericapsular nerve group; SI-FICB: suprainguinal fascia iliaca block; HKSJ: Hartung-Knapp-Sidik-Jonkman

Across the 11 included RCTs (approximately 700 participants analyzed for this endpoint), PENG produced significantly lower immediate pain scores compared with SI-FICB (mean difference: -0.51, 95% CI: -0.74 to -0.28; T=4.97; I²=81%; τ²=0.08). Negative values indicate a reduction in pain, favoring PENG (Figure 5).

Secondary Outcomes

**Figure 6:**
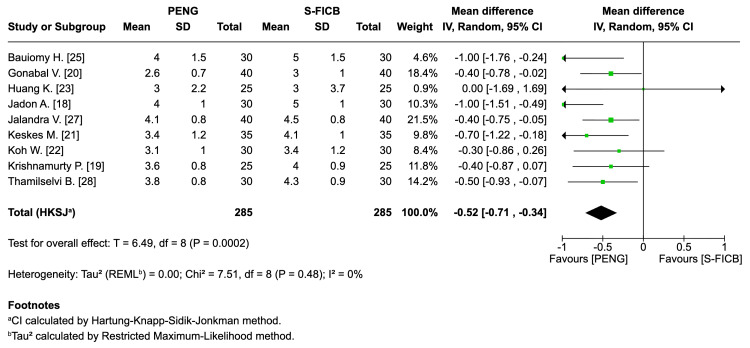
Forest plot - pain on movement within 24 h post-block. REML: random-effects modeling; PENG: pericapsular nerve group; SI-FICB: suprainguinal fascia iliaca block; HKSJ: Hartung-Knapp-Sidik-Jonkman

Pain on movement (≤24 h): Nine trials (approximately 570 participants) demonstrated lower dynamic pain scores in the PENG group (mean difference: -0.52, 95% CI: -0.71 to -0.34; T=6.49; I²=0%), indicating consistent benefit across studies (Figure 6).

**Figure 7:**
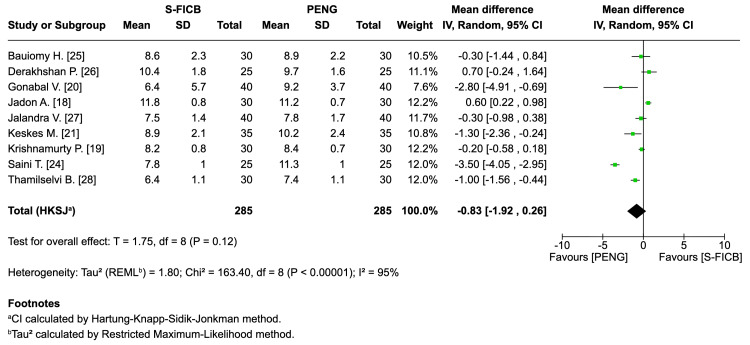
Forest plot - time to first rescue analgesic (hours). REML: random-effects modeling; PENG: pericapsular nerve group; SI-FICB: suprainguinal fascia iliaca block; HKSJ: Hartung-Knapp-Sidik-Jonkman

Time to first rescue analgesic (hours): Nine RCTs (approximately 570 participants) reported no significant difference between groups (mean difference: -0.83 h, 95% CI: -1.92 to 0.26; T=1.75; I²=95%; τ²=1.80) (Figure 7).

**Figure 8:**
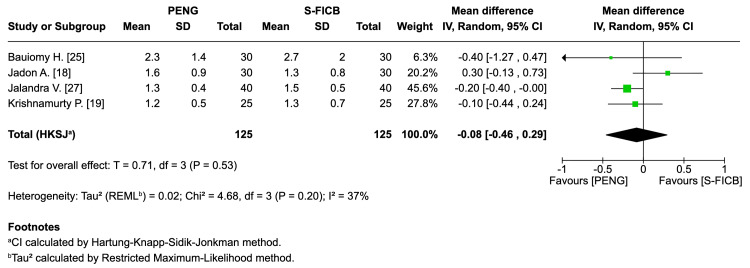
Forest plot - number of rescue doses within 24 h. REML: random-effects modeling; PENG: pericapsular nerve group; SI-FICB: suprainguinal fascia iliaca block; HKSJ: Hartung-Knapp-Sidik-Jonkman

Number of rescue doses (24 h): Four RCTs (approximately 250 participants) showed no significant difference between interventions (mean difference: -0.08 doses, 95% CI: -0.46 to 0.29; T=0.71; I²=37%) (Figure 8).

**Figure 9:**
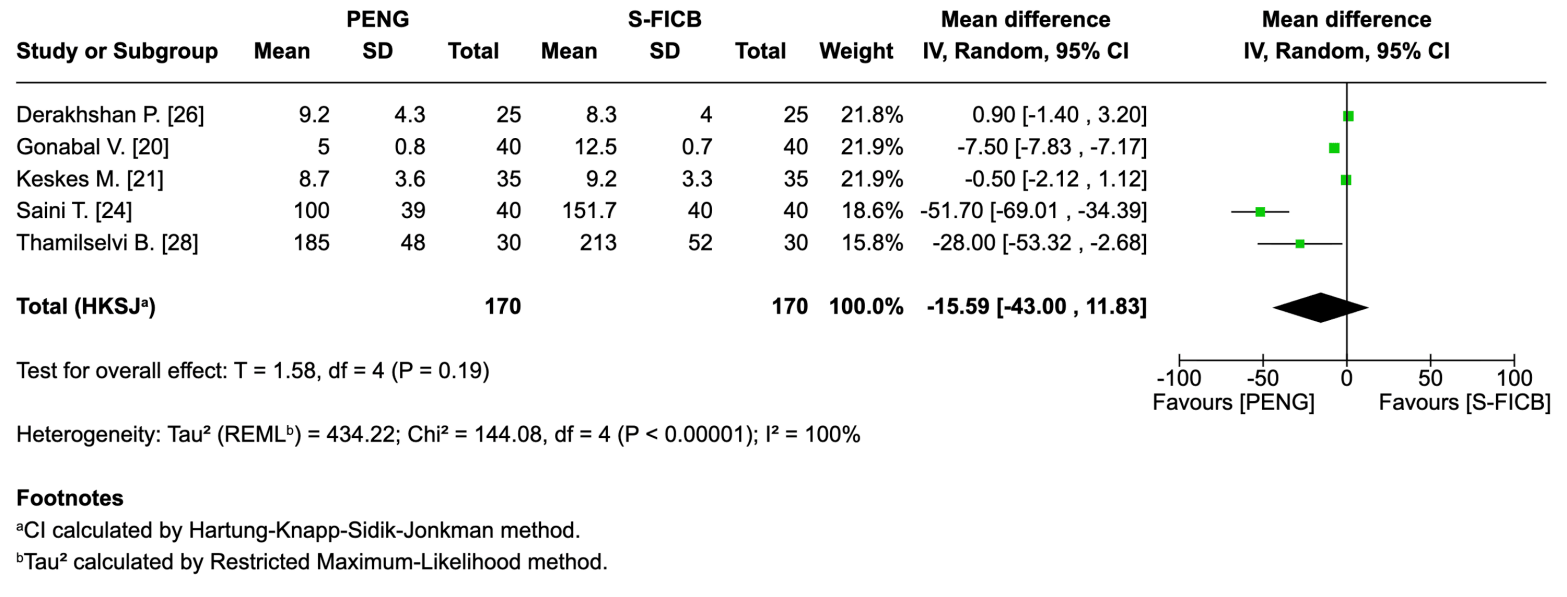
Forest plot - 24 h opioid consumption (oral morphine equivalent, mg). REML: random-effects modeling; PENG: pericapsular nerve group; SI-FICB: suprainguinal fascia iliaca block; HKSJ: Hartung-Knapp-Sidik-Jonkman

Total 24 h opioid consumption (OME, mg): Five RCTs (approximately 340 participants) reported no statistically significant difference in total oral morphine equivalent consumption (mean difference: -15.59 mg, 95% CI: -43.00 to 11.83; T=1.58; I²=100%; τ²=434.22) (Figure 9).

Safety

Adverse events were infrequent across all included trials. Where reported (for example, postoperative nausea or vomiting), event rates were low and comparable between PENG and SI-FICB groups. No trial reported cases of local anesthetic systemic toxicity, vascular injury, or block-site infection.

Discussion

In adults undergoing surgery for hip fracture under neuraxial anesthesia, the pericapsular nerve group (PENG) block provided superior immediate analgesia for spinal positioning, both at rest and on movement, compared with the suprainguinal fascia iliaca compartment block (SI-FICB). There were no significant differences in time to first rescue analgesia, the number of rescue doses, or 24 h opioid consumption. On an 11-point pain scale, the pooled mean difference of approximately 0.5 points represents a modest but potentially meaningful improvement in perioperative comfort, particularly during positioning for spinal anesthesia.

Relation to Prior Evidence and Anatomical Rationale

These findings are consistent with the underlying anatomical rationale that the PENG block selectively targets sensory branches innervating the anterior hip capsule, primarily derived from the femoral and accessory obturator nerves [7]. In contrast, the SI-FICB aims for a broader cephalad spread within the fascia iliaca plane to reach the lumbar plexus. Earlier comparative studies established that suprainguinal modification improves coverage over infrainguinal approaches, but this review demonstrates that PENG achieves greater immediate analgesic efficacy when evaluated in a head-to-head perioperative context. By focusing exclusively on randomized controlled trials comparing PENG with SI-FICB in surgical hip fracture patients, this analysis provides practice-relevant estimates of effectiveness that previous mixed-technique or prehospital meta-analyses could not isolate [4-6].

Clinical Implications

For operating lists prioritizing neuraxial anesthesia, PENG appears advantageous for improving positioning tolerance without increasing early opioid requirements. Incorporating PENG into enhanced recovery protocols may therefore facilitate smoother anesthetic delivery, improved perioperative comfort, and potentially reduced in-tratheatre delays. Where dynamic pain limits early mobilization, the consistent reduction observed with PENG may enhance participation in early physiotherapy and shorten time to ambulation, thereby supporting key elements of postoperative recovery pathways. The motor-sparing characteristics of the PENG block, arising from its selective sensory distribution to the anterior hip capsule, may further facilitate safe mobilization by preserving quadriceps strength and reducing the risk of postoperative instability. This preservation of motor function has important implications in the hip fracture population, in whom compromised mobility and heightened vulnerability to falls can substantially influence recovery trajectories and discharge planning. As a result, the combined analgesic and motor-sparing profile of PENG may contribute not only to improved patient comfort but also to more efficient and safer early rehabilitation.

Heterogeneity and Sources of Variability

The observed heterogeneity, particularly for time-to-rescue and opioid consumption outcomes, likely reflects variations in local anesthetic concentration, total volume, timing of block administration (ward versus theatre), concomitant multimodal analgesic regimens, and differing outcome assessment schedules. Variability in oral morphine-equivalent conversions may also have influenced the pooled estimates. Future investigations should standardize local anesthetic dosing, perioperative timing, and analgesic rescue criteria while ensuring transparent reporting of OME conversion methods to improve data comparability.

Strengths and Limitations

This review’s strengths include a tightly focused clinical question with exclusively perioperative, head-to-head randomized controlled evidence, rigorous meta-analytic methodology using the restricted maximum likelihood model with Hartung-Knapp-Sidik-Jonkman adjustment, and validated transformations of non-parametric data [12,13]. The inclusion of recent multinational RCTs enhances external validity. Limitations include moderate to high heterogeneity for some secondary outcomes, limited power to assess rare adverse events, and inconsistent reporting of functional endpoints such as quadriceps strength, mobilization milestones, and postoperative delirium. The certainty of evidence was rated as moderate for immediate pain outcomes and low for secondary analgesic measures according to GRADE methodology.

Future Research

Future multicenter RCTs should standardize block protocols and outcome definitions, incorporate both pain and motor assessments, and report patient-centered outcomes such as time to mobilization, delirium incidence, and length of stay. Comparative studies evaluating dose-response characteristics, adjuvant use, and imaging-guided refinements of the PENG block are warranted to optimize the balance between analgesia and motor preservation.

## Conclusions

In perioperative randomized controlled trials involving patients undergoing surgery for hip fracture, the pericapsular nerve group (PENG) block provides superior immediate analgesia compared with the suprainguinal fascia iliaca compartment block (SI-FICB), particularly during spinal positioning and early movement. Differences in rescue timing and 24 h opioid consumption were not statistically significant. Incorporating PENG into enhanced recovery pathways may improve comfort and procedural efficiency where expertise is available. Further multicenter, standardized trials are required to clarify its impact on functional recovery and optimize dosing and timing strategies.

## Appendices

Search strategy (PubMed)

(pericapsular nerve group[tiab] OR PENG block[tiab] OR pericapsular block[tiab]) AND (supra-inguinal fascia iliaca[tiab] OR suprainguinal fascia iliaca[tiab] OR fascia-iliaca[tiab] OR fascia iliaca compartment[tiab] OR FICB[tiab]) AND (hip fracture[tiab] OR femoral neck fracture[tiab] OR intertrochanteric fracture[tiab] OR subtrochanteric fracture[tiab]) AND (perioperative[tiab] OR peri-operative[tiab] OR preoperative[tiab] OR intraoperative[tiab] OR spinal anesthesia[tiab] OR anesthesia, spinal[MeSH]) AND (randomized controlled trial[pt] OR controlled clinical trial[pt] OR random*[tiab] OR trial[tiab]) NOT (animals[mh] NOT humans[mh]) NOT ("emergency department"[tiab] OR prehospital[tiab]).

Search strategy (Cochrane)

("pericapsular nerve group" OR "PENG block" OR "pericapsular block") AND ("supra-inguinal fascia iliaca" OR "suprainguinal fascia iliaca" OR "fascia iliaca compartment" OR FICB) AND ("hip fracture" OR "femoral neck fracture" OR "intertrochanteric fracture" OR "subtrochanteric fracture") AND (perioperative OR preoperative OR intraoperative OR "spinal anesthesia") NOT ("emergency department" OR prehospital).

Search strategy (Embase)

('pericapsular nerve group block'/exp OR 'pericapsular nerve group block':ti,ab OR 'PENG block':ti,ab OR 'pericapsular block':ti,ab) AND ('suprainguinal fascia iliaca block'/exp OR 'supra-inguinal fascia iliaca':ti,ab OR 'suprainguinal fascia iliaca':ti,ab OR 'fascia iliaca compartment block':ti,ab OR FICB:ti,ab) AND ('hip fracture'/exp OR 'femoral neck fracture'/exp OR 'intertrochanteric fracture'/exp OR 'subtrochanteric fracture'/exp OR ('hip fracture*' OR 'femoral neck fracture*' OR 'intertrochanteric fracture*' OR 'subtrochanteric fracture*'):ti,ab) AND ('perioperative care'/exp OR perioperativ*:ti,ab OR preoperativ*:ti,ab OR intraoperativ*:ti,ab OR 'spinal anesthesia'/exp) AND ('randomized controlled trial'/exp OR random*:ti,ab OR trial:ti) NOT ('animal'/exp NOT 'human'/exp) NOT ('emergency department':ti,ab OR prehospital:ti,ab)
